# Percutaneous transhepatic obliteration for life-threatening bleeding after endoscopic variceal ligation in a patient with severe esophagogastric varices

**DOI:** 10.1016/j.radcr.2022.10.105

**Published:** 2022-11-30

**Authors:** Fumio Chikamori, Satoshi Ito, Niranjan Sharma

**Affiliations:** a Department of Surgery, Japanese Red Cross Kochi Hospital, 1-4-63-11 Hadaminamimachi, Kochi, 780-8562 Japan; b Department of Radiology, Japanese Red Cross Kochi Hospital, 1-4-63-11 Hadaminamimachi, Kochi, 780-8562 Japan; c Adv Train Gastroint & Organ Transp Surgery, 12 Scotland St, Dunedin, 9016, New Zealand

**Keywords:** Portal hypertension, Splanchnic caput Medusae, Esophagogastric varices, Percutaneous transhepatic obliteration, Partial splenic embolization, Endoscopic variceal ligation

## Abstract

We report a case of life-threatening bleeding after endoscopic variceal ligation (EVL) in a patient with severe esophagogastric varices that was treated by percutaneous transhepatic obliteration (PTO). 3D-CT reconstruction image demonstrated giant esophagogastric varices and gastrorenal shunt. The spleen volume was 813 mL, and the liver volume was 716 mL; giving a spleen/liver volume ratio of 1.1. A strategy of stepwise partial splenic artery embolization (PSE) was employed to control portal venous pressure based on the concept of splanchnic caput Medusae. The S/L ratio improved to 0.3 by stepwise PSE. Subsequently, EVL was performed for esophageal varices, but bleeding occurred afterward, and hemostasis using a Sengstaken-Blakemore tube was attempted. Subsequently, PTO was performed the following day for embolization of the left gastric vein. Gastric varices and gastrorenal shunt were intentionally reserved to avoid portal venous pressure increase. After the procedure, his condition improved. We conclude, in patients with severe esophagogastric varices, prudent management of the splenomegaly and the collateral tracts is necessary.

## Introduction

Endoscopic injection sclerotherapy (EIS) and endoscopic variceal ligation (EVL) are considered the first treatment for esophageal varices [1,2]. Because portal venous pressure is associated with bleeding, it is important to control portal venous pressure to prevent variceal bleeding [3], [4], [5]. We have reported a hybrid procedure that combines endoscopic treatment with partial splenic artery embolization (PSE) for esophagogastric varices [6]. However, in the cases of severe esophagogastric varices with gastrorenal shunt and splenomegaly, optimal therapeutic strategies have not yet been established. We report a case of life-threatening bleeding after endoscopic variceal ligation with severe esophagogastric varices, that was managed by percutaneous transhepatic obliteration (PTO) [7,8].

## Case report

A 64-year-old male with giant esophagogastric varices and splenomegaly due to Wilson's disease was referred to our department. The patient was diagnosed with Wilson's disease with hepatic encephalopathy 5 years ago and was taking zinc acetate hydrate, trientine hydrochloride, penicillamine, lactulose, and kanamycin monosulfate. He underwent ileal conduit surgery for bladder cancer 12 years ago.

On admission, he had jaundice and Grade Ⅰ hepatic encephalopathy according to the West Haven criteria [9]. Laboratory studies revealed hemoglobin 11.9 g/dL (normal range, 13.5-17.4 g/dL), total leukocyte count 1270/µL (3500-8000/µL), platelet count 2.8 × 10^4^/µL (12.3-33.1 × 10^4^/µL), total bilirubin 3.1 mg/dL (0.3-1.3 mg/dL), albumin 3.5 g/dL (3.8-5.0 g/dL), aspartate transaminase 46 U/L (10-32 U/L), alanine transaminase 38 U/L (5-27 U/L), prothrombin time 50% (70%-130%), Mac-2 binding protein glycosylated isomers 10.93 COI (2+) (<1.00), serum ammonia 251 µg/dL (12-66 µg/dL), serum zinc 86µg/dL (80-110 µg/dL), ceruloplasmin level 2.7 mg/dL (21.0-37.0 mg/dL), and serum copper 14 µg/dL (70-132 µg/dL). The retention rate of indocyanine green at 15 minutes was 46% (<10%). The Child-Pugh score was 10 and the class was C. Hepatitis B surface antigen and hepatitis C virus antibody were negative.

**Fig. 1:**
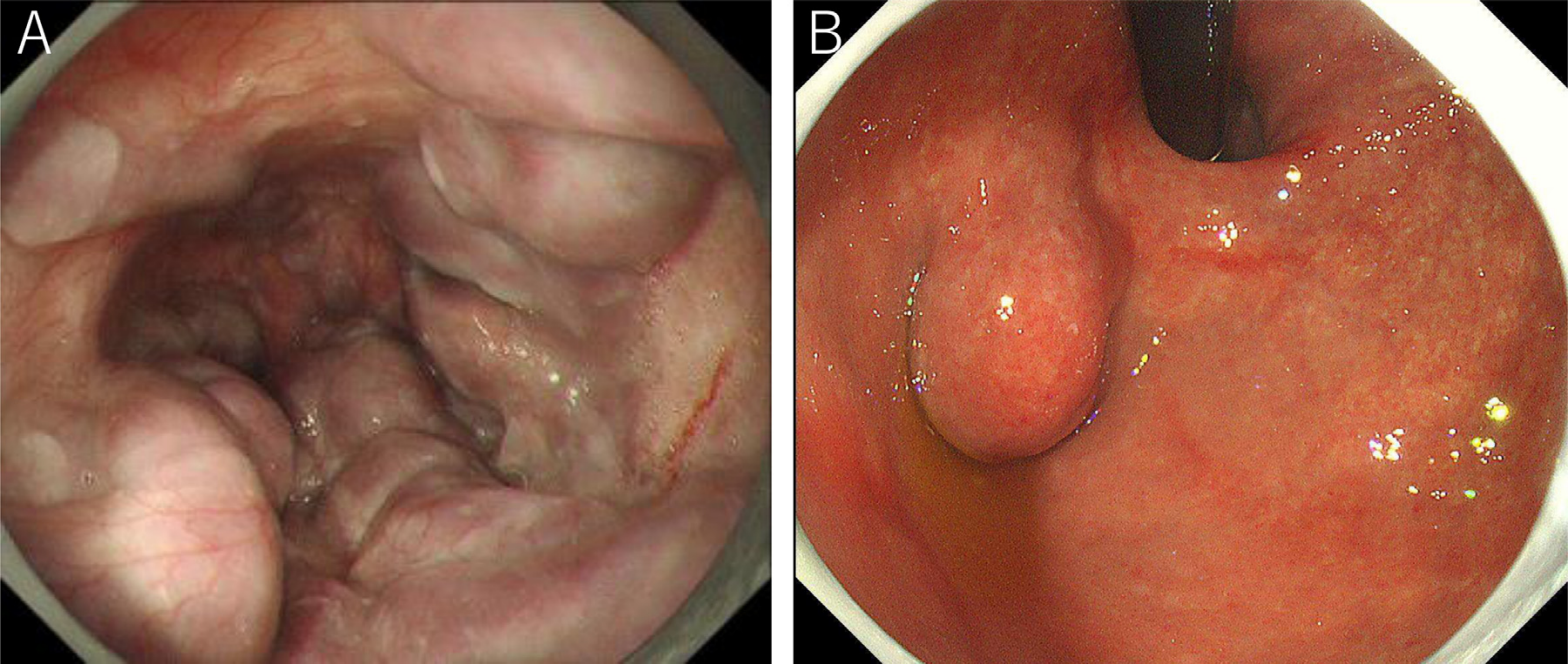
Endoscopic picture before treatment. (A) Endoscopic picture shows markedly enlarged nodular-shaped (F3) esophageal varices. (B) Endoscopic picture shows moderately enlarged (F2) gastric varices.

**Fig. 2:**
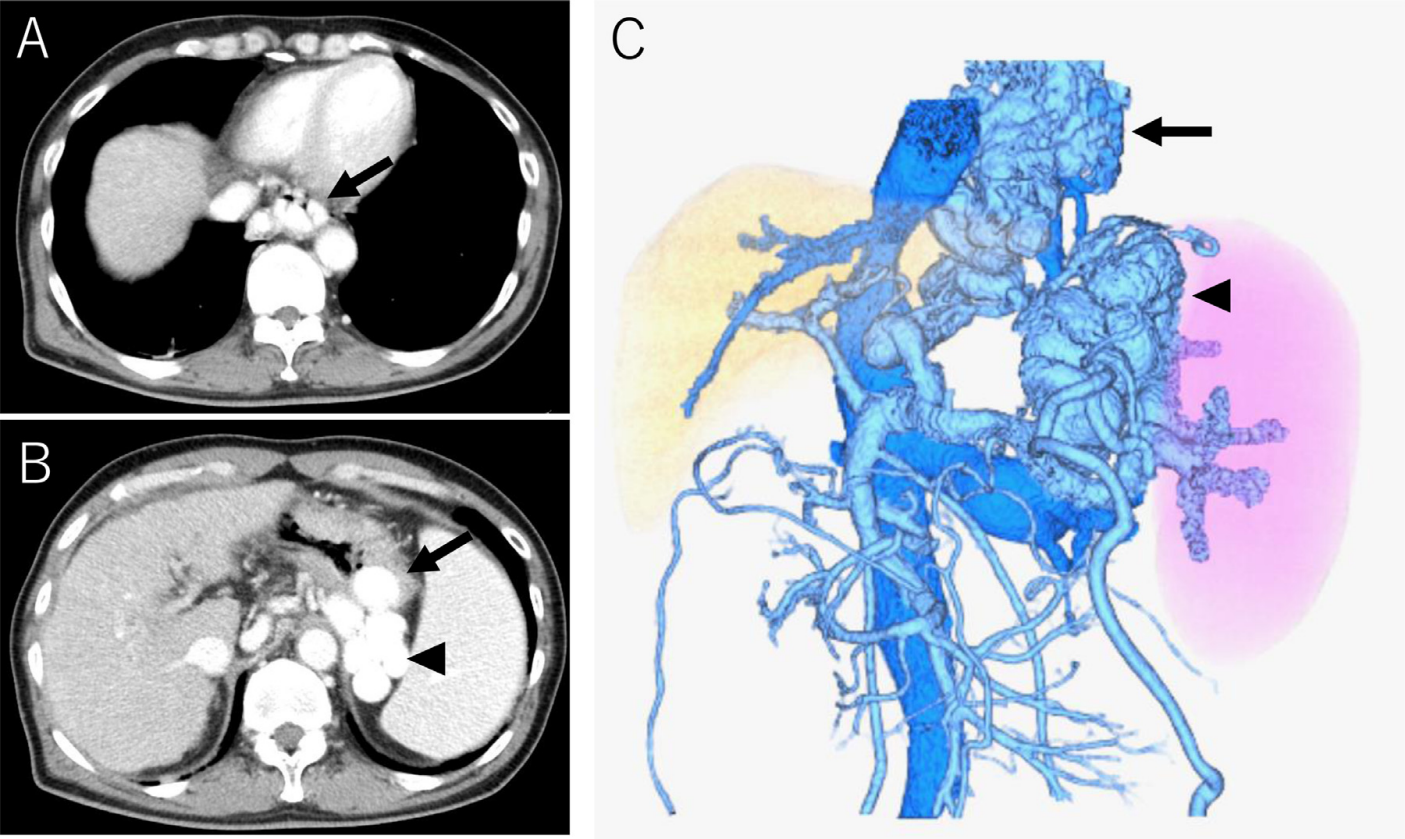
Contrast-enhanced CT and 3D-CT reconstruction image before treatment. (A) Contrast-enhanced CT before treatment shows giant esophageal varices (black arrow). (B) Contrast-enhanced CT before treatment shows gastric varices (arrow) and giant gastrorenal shunt (arrowhead). (C) 3D-CT reconstruction image before treatment shows giant esophageal varices (arrow) and gastrorenal shunt (arrowhead) with severe splenomegaly. The S/L ratio is 1.1.

Endoscopy confirmed markedly enlarged nodular-shaped esophageal varices (F3) and moderately enlarged gastric varices (F2) (Figs. 1A and B) [10]. Abdominal ultrasonography and contrast-enhanced CT revealed severe splenomegaly and a giant gastrorenal shunt (Figs. 2A and B). 3D-CT reconstruction image demonstrated that the giant esophageal varices were supplied by the left gastric vein. The gastric varices were supplied by the short gastric vein and drained into the left renal vein. The portal vein was narrowed. The spleen volume was 813 mL, and the liver volume was 716 mL; giving a spleen/liver volume ratio (S/L ratio ) [11] of 1.1 (Fig. 2C). According to the “splanchnic caput Medusae” concept [12], the enlarged spleen was regarded as her face, and the esophagogastric varices as her snake hairs. This case had giant esophageal varices despite the presence of a significant-sized gastrorenal shunt. Esophageal varices were significantly distended and were near to rupture causing sudden death. He was considered to have severe portal hypertension. Initial control of portal venous pressure was thought to be necessary to prevent variceal bleeding. A stepwise PSE [12] was implemented.

**Fig. 3:**
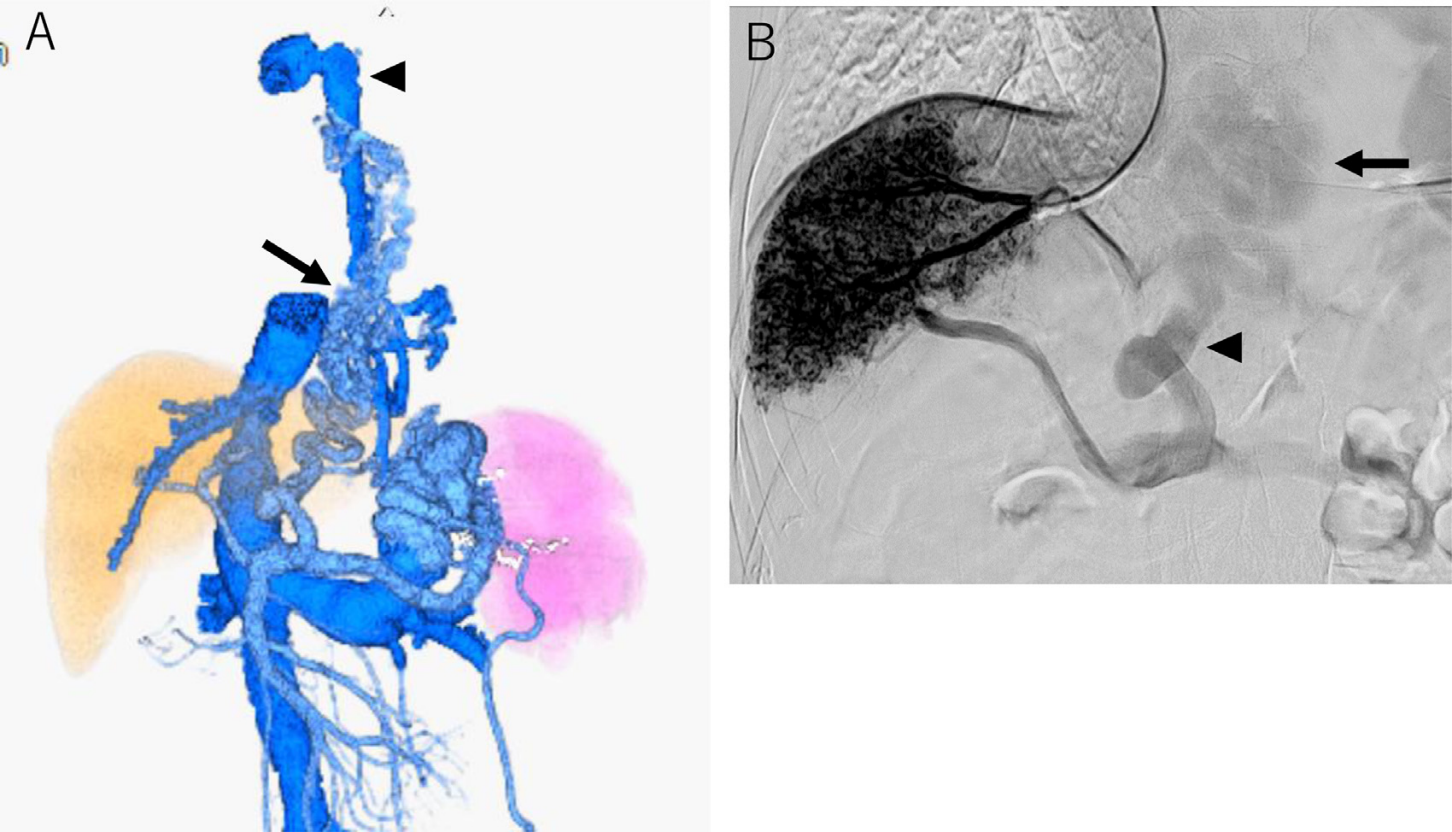
3D-CT reconstruction image and retrograde hepatic venography before EVL. (A) 3D-CT reconstruction image 3 months after the second PSE shows esophageal varices (arrow) and spleen reduced size. The esophageal variceal blood is draining into the azygos vein (arrowhead). (B) Retrograde hepatic venography shows a large left gastric vein (arrowhead) and esophageal varices (arrow).

The portal phase of superior mesenteric arteriography revealed the hepatofugal flow of the left gastric vein and gastrorenal shunt. The S/L ratio changed to 0.9 after the first PSE. Two months later, the second PSE was performed, and the S/L ratio improved to 0.3 (Fig. 3 A). Wedged hepatic venous pressure (WHVP)/hepatic venous pressure gradient (HVPG) changed from 19/16 to 19/11 mmHg by the second PSE. The platelet count also increased to 7.8 × 10^4^/µL.

**Fig. 4:**
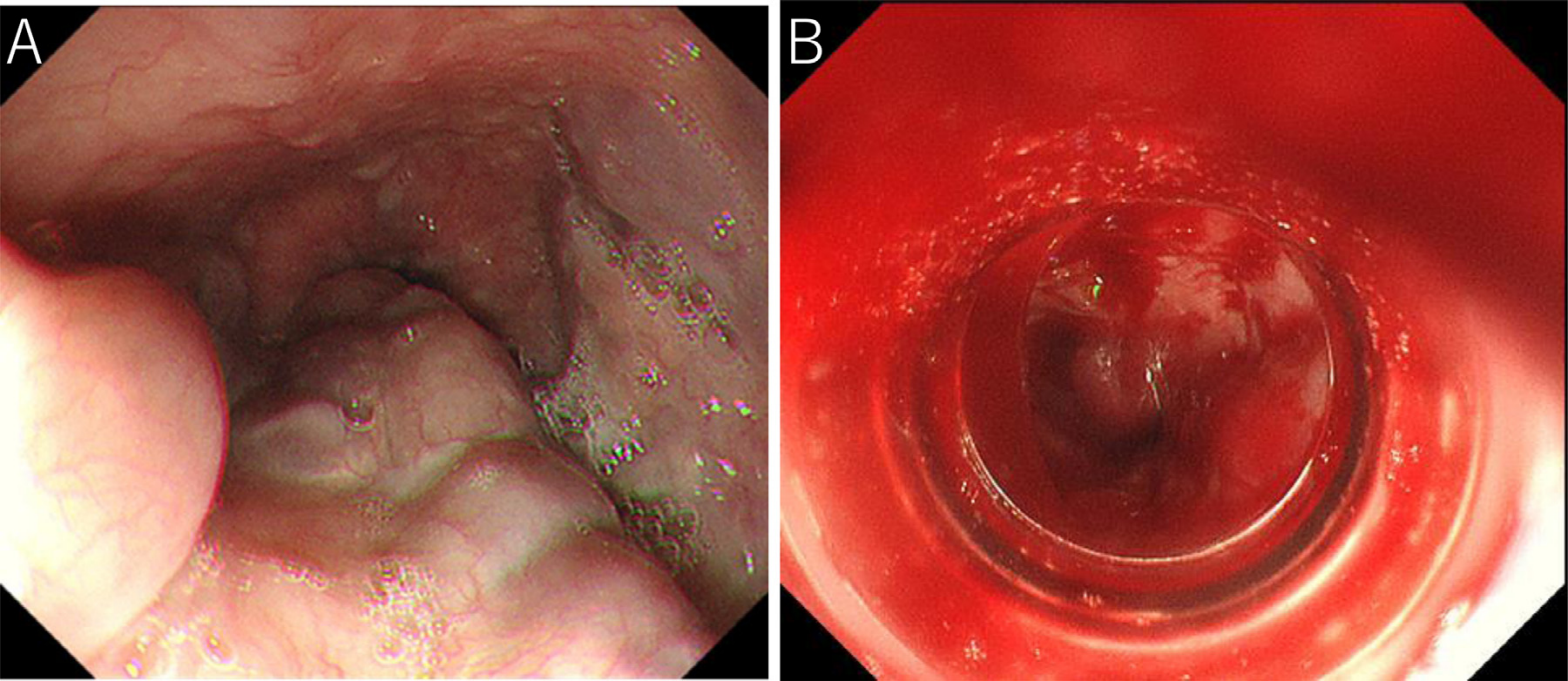
Endoscopic picture before and after EVL. (A) Endoscopic picture before EVL shows moderately enlarged beads-like (F2) esophageal varices, reduced in size by stepwise PSE. (B) Endoscopic picture after EVL shows oozing hemorrhage.

**Fig. 5:**
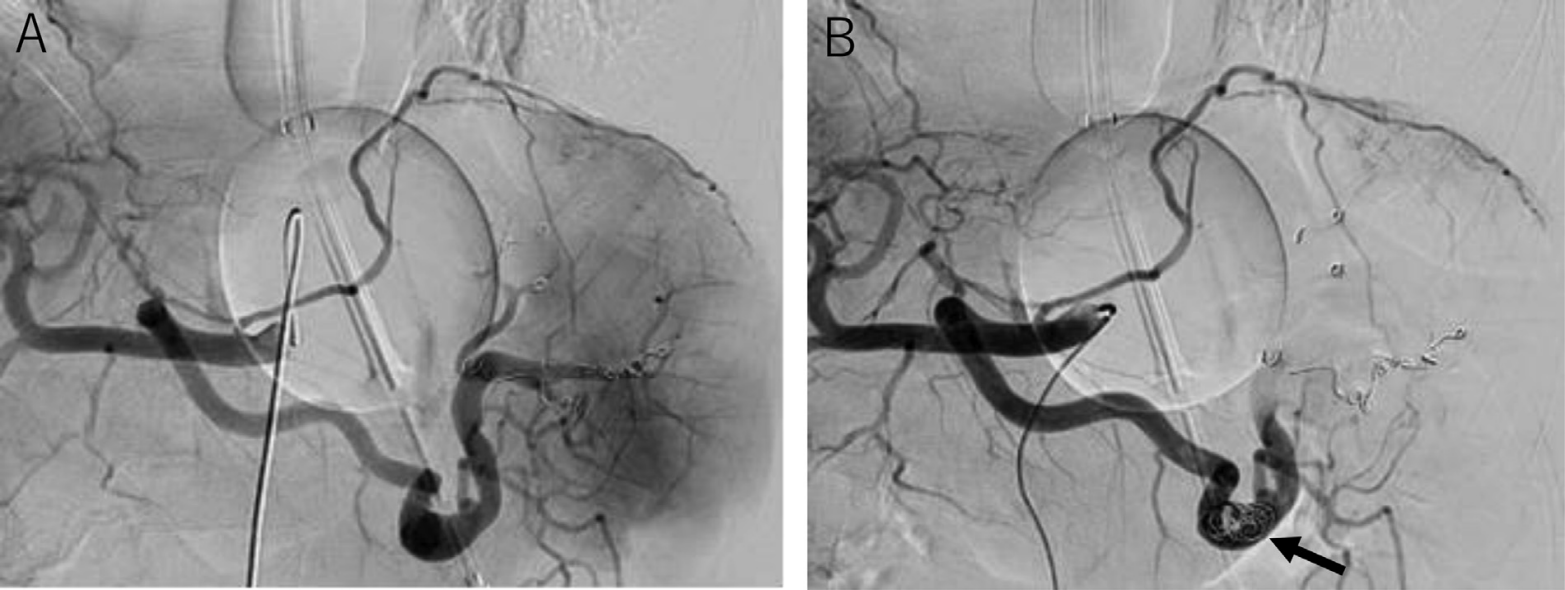
Additional splenic artery embolization before PTO. (A) Celiac arteriogram before additional splenic artery embolization. (B) Celiac arteriogram after additional splenic artery embolization. Embolized microcoils are indicated by an arrow.

**Fig. 6:**
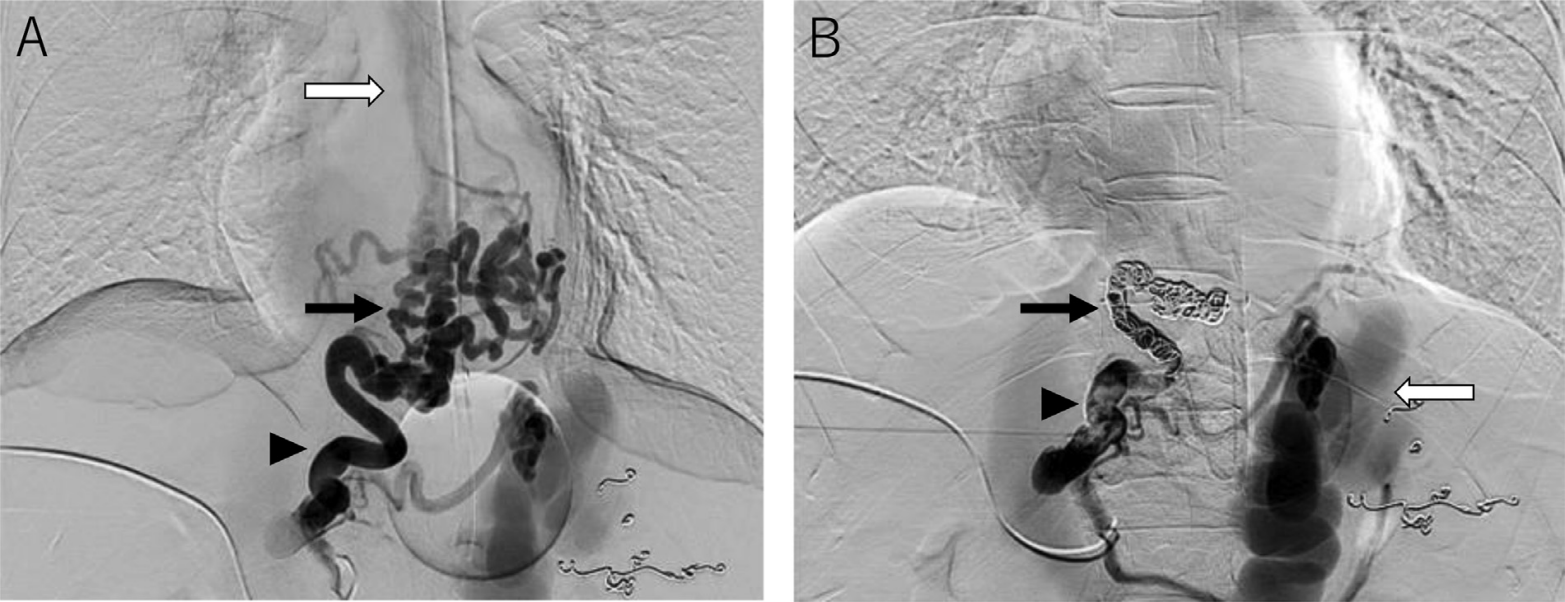
PTO. (A) Percutaneous transhepatic left gastric venography shows the left gastric vein (arrowhead), paraesophageal vein (black arrow), and azygos vein (white arrow). (B) The left gastric vein is embolized using microcoils (black arrow) and 25% NBCA (arrowhead). The gastrorenal shunt (white arrow) is not affected.

**Fig. 7:**
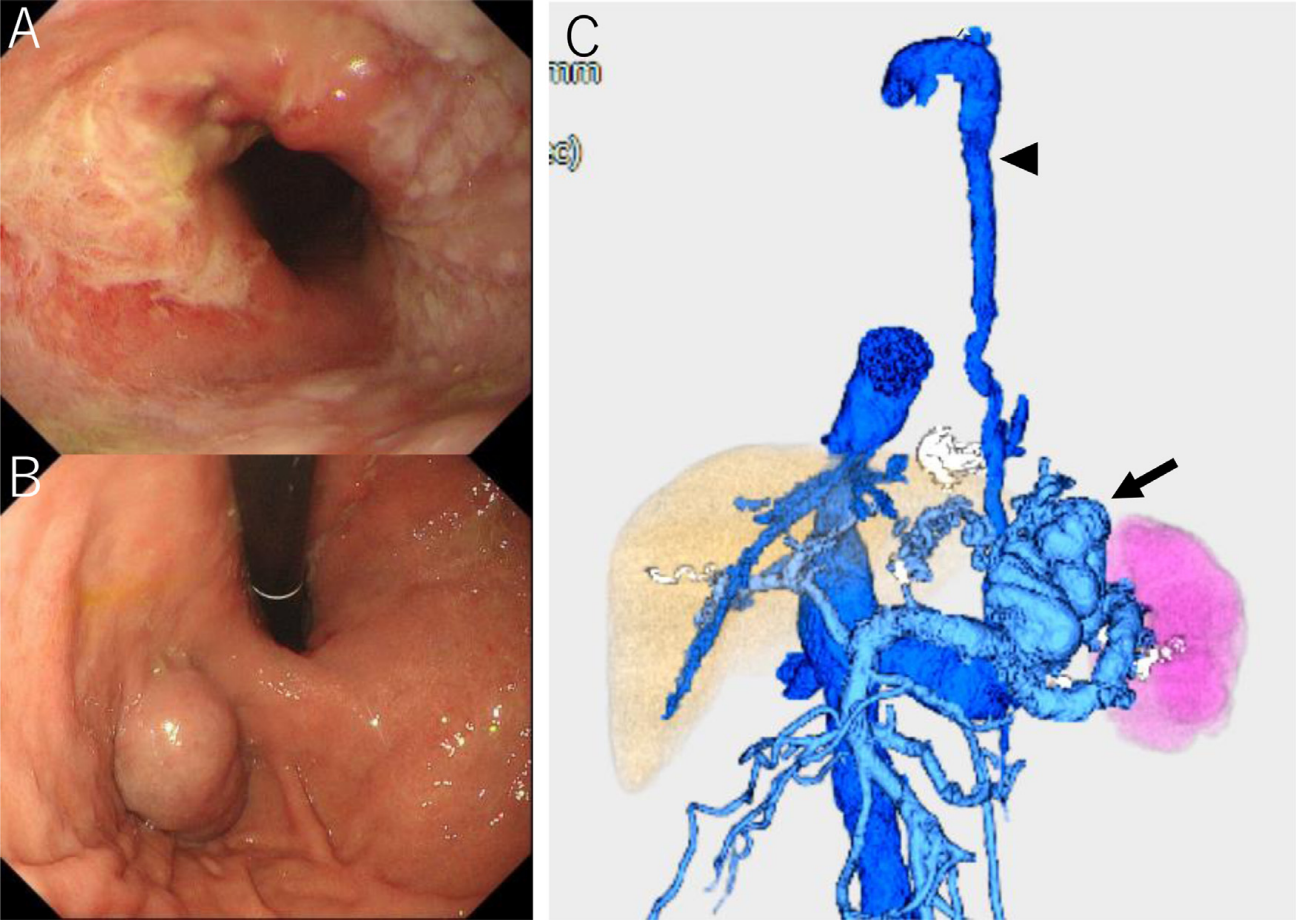
Endoscopic picture and 3D-CT reconstruction image after treatment. (A) Endoscopic picture 3 weeks after PTO shows disappearance of esophageal varices. (B) Endoscopic picture 3 weeks after PTO shows no change of gastric varices. (C) 3D-CT reconstruction image 30 months after PTO shows the disappearance of esophageal varices. The gastrorenal shunt and azygos vein are not affected. The S/L ratio is 0.3.

Three months after the second PSE, endoscopic injection sclerotherapy with ligation (EISL) [13,14] was planned, but was abandoned due to unstable liver function and the general condition of the patient. At this time, WHVP/HVPG had changed again to 26/16 mmHg on hepatic venous catheterization. Retrograde hepatic venography revealed the development of the hepatofugal left gastric vein and esophageal varices (Fig. 3B). Four months after the second PSE, the patient's condition became stable, and the esophageal varices were reduced to moderately enlarged, beads-like varices (F2) (Fig. 4A), so EVL was attempted. However, the varicose vein next to the EVL site was swollen one after another, and band ligations were repeated. This required 17 ligations finally, but oozing hemorrhage was observed (Fig. 4B). As subsequent massive bleeding was suspected, a Sengstaken-Blakemore tube (SB tube) was prophylactically inserted. As suspected, 9 hours later, the patient vomited blood. It was managed by SB tube balloon inflation. Despite blood transfusion and IV fluid, the patient's vital signs were not stabilized attracting interventional radiology on the following day. Initially, splenic artery embolization was performed to control portal venous pressure (Figs. 5A and B). Second, PTO was performed to embolize the left gastric vein, which was the main blood supply route of the esophageal varices. The left gastric vein was embolized using microcoils and 25% n-butyl-2-cyanoacrylate (NBCA) (Histoacryl) with ethyl ester of iodinated poppy-seed oil fatty acid (Lipiodol) 2 mL in total (Figs. 6A and B). We intentionally preserved gastric varices and gastrorenal shunt, to avoid portal venous pressure increase. The portal venous pressure changed from 14 to 15 mmHg by PTO. After the procedure, the bleeding was controlled. The initial worsening of hepatic encephalopathy gradually improved with conservative therapy, which included the administration of branched-chain amino acids. 3D-CT reconstruction image 5 days after PTO demonstrated that the esophageal varices disappeared, and the gastric varices with gastrorenal shunt were not affected. Endoscopy 3 weeks after PTO revealed the disappearance of esophageal varices (Figs. 7A and B). The patient was discharged on the 50th hospital day.

Thirty months after PTO, the platelet count was 8.7 × 10^4^/µL, and the Child-Pugh score improved to 8 (class B). Endoscopic examination showed no change in gastric varices, but no recurrence of esophageal varices. 3D-CT reconstruction image demonstrated no recurrence of esophageal varices. The spleen volume was 194 mL, and the liver volume was 694 mL. The S/L ratio was maintained at 0.3 (Fig. 7C).

## Discussion

We reported a case of life-threatening bleeding after EVL in a patient with severe esophagogastric varices, that was managed by PTO [7,8]. There were 3 important issues in this case: 1. What is the pathophysiology of severe esophagogastric varices with splenomegaly and giant gastrorenal shunt? 2. Is there an indication for prophylactic treatment for severe esophagogastric varices with gastrorenal shunt? 3. What is the best strategy for the treatment of severe esophagogastric varices?

The presence of a gastrorenal shunt usually contributes to portal pressure decompression. When esophageal varices coexist with gastrorenal shunt, they are usually small in size [15,16]. However, in this case, esophageal varices were markedly enlarged. This was due to severe splenomegaly, liver atrophy, and a markedly increased S/L ratio. We consider that due to the marked increase in splenic arteriovenous blood flow associated with severe splenomegaly, the portal pressure could not be buffered by the gastrorenal shunt alone; leading to the formation of giant esophageal varices. This condition was a severe transformation in portal hypertension. Since 2020, we have proposed a new concept: “splanchnic caput Medusae” in which the spleen is her face and portal collateral pathways are her snake hairs [12]. This case can be said to be the strongest monster Medusae state.

In the Baveno VI consensus report [17], either a nonselective beta-blocker or endoscopic band ligation is recommended for the prevention of the first variceal bleeding of medium or large varices. Moreover, in this case, the esophageal varices were very tense and could have caused sudden death if they ruptured. Therefore, prophylactic treatment was planned.

In the Baveno VI Consensus report [17], the choice of treatment should be based on local resources and expertise, patient preference and characteristics, contraindications, and adverse events. In Japan, prophylactic EIS by skillful endoscopists is widely performed [18]. However, EIS using ethanolamine oleate is contraindicated in patients with severe hepatic disorders (Child-Pugh C, total bilirubin 4.0 mg/dL or more), where alternative EVL can be performed. In cases with severe platelet depletion (2.0 × 10^4^/µL or less), endoscopic treatment is contraindicated. In this case, total bilirubin was 3.1 mg/dL, and platelet count was 2.8 × 10^4^/µL. This case was considered within the threshold for the therapeutic indication for EIS. Therefore, we first attempted to correct splenic circulation and improve platelet counts by stepwise PSE. As a result, the S/L ratio reduced from 1.1 to 0.3, and HVPG also reduced from 16 to 11 mmHg.

After the satisfactory outcome as outlined above, EISL was planned. However, the procedure was abandoned because liver function and general condition were noted unstable and HVPG increased again. After 1 month of conservative management, liver function and general condition had stabilized, and esophageal varices had decreased to medium size. Thus, EVL was considered feasible. However, blood flow blockage by EVL caused varicose veins to swell one after another, requiring several band ligations to stabilize the varicose veins. Despite careful preparation, bleeding occurred after EVL, suggesting that EISL should have been selected with more follow-up. Although the EVL procedure is simple, it should be recognized that it can cause fatal bleeding. Early dislodgement of the rubber band can lead to bleeding from the ulcer before the occlusion of the varicose vein by a mature thrombus. Reports of this kind of serious complication are limited [19], [20], [21].

Transjugular intrahepatic portosystemic shunt is recommended for bleeding esophageal varices that cannot be controlled endoscopically [22]. However, it was not applied in this case due to the presence of a giant gastrorenal shunt. We instituted PTO as a rescue procedure for life-threatening bleeding after EVL in which the left gastric vein, which is the main blood supplier; was embolized.

Indications of PTO are limited to special cases such as intractable varices or ectopic varices [8]. Based upon the clinical findings, this case falls within a special cases category as described above. Preservation of the gastrorenal shunt was considered essential because PTO increases portal venous pressure. Furthermore, additional splenic artery embolization was performed before PTO. It was considered that embolization of the splenic artery may have contributed to portal pressure reduction. The recurrence of esophageal varices due to recanalization of the blood supply is one of the disadvantages of PTO [7]. The patient did not have a recurrence of esophageal varices even 30 months after PTO. The control of inflow by reduction of splenic volume and the effect of portal decompression by gastrorenal shunt may have contributed to the prevention of recurrence. Although this patient had mild encephalopathy, the gastrorenal shunt was intentionally preserved because treatment of variceal bleeding was the priority in this case. In the future, when encephalopathy due to gastrorenal shunt becomes uncontrollable, shunt embolization may be considered depending on HVPG and liver function.

The severe esophagogastric varices, which are at the limit of therapeutic indications, are likely to cause complications [21], and it is important to treat the splenomegaly and the collateral tracts in a well-balanced manner. We conclude that PTO is one of the useful rescue procedures for life-threatening bleeding after EVL for severe esophagogastric varices.

## Patient consent

Written informed consent was obtained from the patient for publication of this case report and accompanying images.

## References

[1] Obara K., Obara K (2019). Clinical investigation of portal hypertension.

[2] Takase Y, Shibuya S, Chikamori F, Orii K, Iwasaki Y. (1990). Recurrence factors studied by percutaneous transhepatic portography before and after endoscopic sclerotherapy for esophageal varices. Hepatology.

[3] Garcia-Tsao G, Abraldes JG, Berzigotti A, Bosch J. (2017). Portal hypertensive bleeding in cirrhosis: risk stratification, diagnosis, and management: 2016 practice guidance by the American Association for the Study of Liver Diseases. Hepatology.

[4] Albilllos A, Garcia-Tsao G. (2011). Classification of cirrhosis: the clinical use of HVPG measurements. Dis Markers.

[5] Bosch J, Garcia-Pagan JC. (2003). Prevention of variceal rebleeding. Lancet.

[6] Chikamori F, Maeda A, Sharma N. (2022). An emergency hybrid procedure that combines endoscopic treatment with partial splenic embolization for bleeding esophagogastric varices. Radiol Case Rep.

[7] Lunderquist A, Simert G, Tylén U, Vang J. (1977). Follow-up of patients with portal hypertension and esophageal varices treated with percutaneous obliteration of gastric coronary vein. Radiology.

[8] Chikamori F, Kuniyoshi N, Kagiyama S, Kawashima T, Shibuya S, Takase Y. (2007). Role of percutaneous transhepatic obliteration for special types of varices with portal hypertension. Abdomin Imaging.

[9] Vilstrup H, Amodio P, Bajaj J, Cordoba J, Ferenci P, Mullen KD (2014). Hepatic encephalopathy in chronic liver disease: 2014 practice guideline by the American Association for the Study of Liver Diseases and the European Association for the Study of the Liver. Hepatology.

[10] Tajiri T, Yoshida H, Obara K, Onji M, Kage M, Kitano S (2010). General rules for recording endoscopic findings of esophagogastric varices (2nd edition). Dig Endosc.

[11] Chikamori F, Nishida S, Selvaggi G, Tryphonopoulos P, Moon JI, Levi DM (2010). Effect of liver transplantation on spleen size, collateral veins, and platelet counts. World J Surg.

[12] Chikamori F, Sharma N, Ito S, Mizobuchi K, Ueta K, Takasugi H (2020). Stepwise partial splenic embolization for portal hypertension based on a new concept: Splanchnic caput Medusae. Radiol Case Rep.

[13] Nishikawa Y, Y Hosokawa Y, Doi T, Shima S, Miyoshi M, Ohnishi T (1995). Simultaneous combination of endoscopic sclerotherapy and endoscopic ligation for esophageal varices. Gastrointest Endosc.

[14] Chikamori F, Kanazawa S, Sharma N. (2022). Verification of thrombus formation just after endoscopic injection sclerotherapy with ligation for esophagogastric varices by venous phase of left gastric arteriography. Radiol Case Rep.

[15] Watanabe K, Kimura K, Matsutani S, Ohto M, Okuda K. (1988). Portal hemodynamics in patients with gastric varices. A study in 230 patients with esophageal and/or gastric varices using portal vein catheterization. Gastroenterology.

[16] Chikamori F, Kuniyoshi N, Shibuya S, Takase Y (2001). Correlation between endoscopic and angiographic findings in patients with esophageal and isolated gastric varices. Dig Surg.

[17] de Franchis R (2015). Baveno VI Faculty. Expanding consensus in portal hypertension: report of the Baveno VI Consensus Workshop: Stratifying risk and individualizing care for portal hypertension. J Hepatol.

[18] Gotoh Y, Iwakiri R, Sakata Y, Koyama T, Noda T, Matsunaga C (1999). Evaluation of endoscopic variceal ligation in prophylactic therapy for bleeding of oesophageal varices: a prospective, controlled trial compared with endoscopic injection sclerotherapy. J Gastroenterol Hepatol.

[19] Mishin I, Dolghii A. (2005). Early spontaneous slippage of rubber bands with fatal bleeding: a rare complication of endoscopic variceal ligation. Endoscopy.

[20] Toyoda H, Fukuda Y, Katano Y, Ebata M, Nagano K, Morita K (2001). Fatal bleeding from a residual vein at the esophageal ulcer base after successful endoscopic variceal ligation. J Clin Gastroenterol.

[21] Van Vlierberghe H, De Vos M, Hautekeete M, Elewaut A. (1999). Severe bleeding following endoscopic variceal ligation: should EVL be avoided in Child C patients?. Acta Gastroenterol Belg.

[22] Satapathy SK, Sanyal AJ. (2014). Nonendoscopic management strategies for acute esophagogastric variceal bleeding. Gastroenterol Clin North Am.

